# Emergency treatment delay and underdosing are associated with poor outcome in non‐convulsive status epilepticus

**DOI:** 10.1002/epi.70414

**Published:** 2026-07-25

**Authors:** Francesco Misirocchi, Alice Ballabeni, Maddalena Frapporti, Alessandro Borgna, Lucia Zinno, Stefano Meletti, Irene Florindo

**Affiliations:** ^1^ Unit of Neurology University Hospital of Parma Parma Italy; ^2^ Unit of Neurology, Department of Medicine and Surgery University of Parma Parma Italy

**Keywords:** Anti‐seizure medication, benzodiazepine, emergency department, NCSE, outcome

## Abstract

Nonconvulsive status epilepticus (NCSE) is difficult to recognize in the emergency department (ED), where onset is often unknown and treatment may be delayed. To what extent treatment timing and dose adequacy are associated with outcome in NCSE remains debated. In this single‐center electroencephalography (EEG)–based study, adults with NCSE diagnosed in the ED were included. Timing was measured using ED arrival as a temporal anchor. Outcomes were failure to return to baseline at discharge and in‐hospital mortality. Among 74 patients (mean age 66.8), 38 (51.4%) failed to return to baseline and 14 (18.9%) died. ED benzodiazepines (BDZs) were administered in 43 patients (58.1%) and anti‐seizure medications (ASMs) in 71 (95.9%). Median times to BDZ and ASM administration were 184 and 223 min, respectively. After adjustment for Status Epilepticus Severity Score (STESS), potentially fatal etiology, and premorbid modified Rankin Scale score, time to ASM (odds ratio [OR]: 1.11 per hour; 95% confidence interval [CI]: 1.01–1.22; *p* = .037) and ASM underdosing (OR: 3.43; 95% CI: 1.10–10.68; *p* = .034) were independently associated with failure to return to baseline, with time to BDZ showing a non‐significant trend. BDZ underdosing was not associated with functional outcome, and no treatment variable was associated with mortality. These findings support early EEG‐based recognition and rapid, adequately dosed ASM treatment as key targets in ED‐NCSE management.

**Key points**

1. Delayed and underdosed anti‐seizure medication were associated with failure to return to baseline.
2. Benzodiazepine use was not independently associated with outcomes.
3. No treatment variables were independently associated with in‐hospital mortality.
4. Emergency department (ED) door‐to‐treatment is a pragmatic and feasible anchor to evaluate treatment adequacy.
5. Our findings support rapid electroencephalography (EEG)–based pathways in ED‐NCSE (nonconvulsive status epilepticus).

## 1 INTRODUCTION

Nonconvulsive status epilepticus (NCSE) is not uncommon in the emergency department (ED), but its presentation is variable and often nonspecific, including failure to return to baseline after a motor seizure, language disturbance, or fluctuating impairment of consciousness.^1^ Patients may therefore be misclassified as having a postictal state, stroke, or encephalopathy.^2^ ED diagnostic pathways are usually driven by neuroimaging, whereas reliable bedside predictors of patients requiring emergent electroencephalography (EEG) remain limited. After non‐explanatory imaging, NCSE recognition depends on neurological suspicion and EEG availability, with potential treatment delay.^3^, ^4^

NCSE treatment is supported by limited evidence, with current recommendations largely extrapolated from convulsive status epilepticus (CSE) management.^5^, ^6^ However, NCSE differs from CSE in patient profile, clinical expression, etiology, and the frequent absence of a reliable seizure‐onset time.^7^

Whether treatment delay and reduced benzodiazepine (BDZ) and anti‐seizure medication (ASM) loading doses influence prognosis in NCSE remains uncertain.^5^ We therefore assessed the association between treatment timing, loading dose, and outcome, in ED‐NCSE, using ED arrival as a reproducible temporal anchor.

## 2 METHODS

### 2.1 Study design and population

We conducted a single‐center ambispective study nested within an EEG‐based SE registry at the University Hospital of Parma, Italy. Data were collected retrospectively until October 2023, and prospectively thereafter. Consecutive adults with non‐hypoxic NCSE diagnosed between January 2020 and December 2025 were screened. Patients were eligible when NCSE was diagnosed within 24 h of ED arrival while they were still located in the ED. Patients with SE with prominent motor features in the ED were excluded; those with possible prehospital seizures were included if no ongoing motor SE was documented after ED arrival. The study followed the Declaration of Helsinki and STrengthening the Reporting of OBservational studies in Epidemiology (STROBE) recommendations.

### 2.2 Data collection and outcomes

All ED‐NCSE diagnoses fulfilled Salzburg Consensus criteria, including definite NCSE and ictal‐interictal continuum (IIC) patterns associated with electroclinical improvement after anti‐seizure treatment.^6^, ^8^ All EEG recordings were interpreted by experienced certified neurophysiologists (F.M., I.F., and L.Z.), with consensus review in uncertain cases. EEG is available 24/7 in our ED and is requested by the neurology consultant after bedside evaluation or discussion with the ED physician. We extracted patient characteristics, NCSE features, diagnostic pathway, treatments, and timing from ED charts and EEG reports. Only BDZs administered in the ED before any ASM (first‐line ED‐BDZs) were considered for analyses; prehospital BDZ administration was recorded separately. Severity was assessed using the Status Epilepticus Severity Score (STESS) and the Epidemiology‐Based Mortality Score in Status Epilepticus (EMSE).^9^, ^10^ Etiology was classified according to International League Against Epilepsy (ILAE) categories and further grouped as potentially fatal or non‐fatal, as reported previously.^9^, ^11^

BDZ and ASM treatments were classified as underdosed when the administered loading dose was lower than two‐thirds of the recommended dose according to current Italian and European SE guidelines (reference doses and thresholds are reported in Table S1).^5^, ^12^, ^13^

Outcomes were failure to return to baseline at discharge, defined as an increase in modified Rankin Scale (mRS) score of at least 1 point, and in‐hospital mortality.

### 2.3 Statistical analysis

Categorical variables are reported as counts and percentages; continuous variables as mean ± standard deviation (SD) or median with interquartile range (IQR). Group comparisons used Fisher exact, Pearson chi‐square, or Mann–Whitney *U* tests, as appropriate. Multivariable logistic regression models were fitted separately for each treatment exposure and outcome and adjusted for STESS, potentially fatal etiology, and premorbid mRS. Odds ratios (ORs) are reported with 95% confidence intervals (CIs). Analyses were performed with Jamovi, version 2.6.45. Statistical significance was set at *p* < .05.

## 3 RESULTS

### 3.1 Patients and NCSE features

Among 194 NCSE patients, 74 patients fulfilled the inclusion criteria (mean age ± SD 66.8 ± 17.6 years; 32 male, 43.2%). Etiology was acute in 31 patients (41.9%), remote in 18 (24.3%), progressive in 23 (31.1%), and within a defined SE syndrome in 2 (2.7%); 21 episodes (28.4%) had potentially fatal etiology.

Possible prehospital seizure was documented in 28 patients (37.8%), prehospital BDZs were given in 11 (14.9%), and stroke code was activated before ED arrival in 24 (32.4%).

On ED assessment, 17 patients (23.0%) had an acute focal deficit other than language disturbance, 13 (17.6%) had language disturbance, and 33 (44.6%) had impaired consciousness. Brain computed tomography (CT) was performed in 71 patients (95.9%), CT angiography in 20 (27.0%), and CT perfusion in 5 (6.8%). Median time from ED arrival to CT was 74 min (IQR 46–197), whereas median time to EEG was 221 min (IQR 80–668). ED‐BDZs were administered in 43 patients (58.1%) after a median of 184 min (IQR 93–554). ASMs were administered in 71 patients (95.9%) after a median of 223 min (IQR 147–533), and were used as first‐line ED treatment in 31 (41.9%). BDZ underdosing occurred in 13 of 43 patients (30.2%), and ASM underdosing in 32 of 71 (45.1%). At discharge, 38 patients (51.4%) failed to return to baseline and 14 (18.9%) died.

### 3.2 Univariate analysis

Univariate associations are shown in Table 1. Higher STESS and EMSE were associated with both failure to return to baseline and in‐hospital mortality. Older age, potentially fatal etiology, and language disturbance were associated with failure to return to baseline, whereas higher premorbid mRS was associated with mortality.

**TABLE 1:** Univariate associations with failure to return to baseline and in‐hospital mortality.

Variable	Returned to baseline		*p*	In‐hospital outcome		*p*
	Yes (*n* = 36)	No (*n* = 38)		Survival (*n* = 60)	Death (*n* = 14)	
Age, years, median [IQR]	66 [56–76]	73 [64–81]	.024	70 [58–77]	76 [66–83]	.070
Premorbid mRS, median [IQR]	2 [1–3]	3 [2–4]	.151	2 [1–4]	3 [3–4]	.007
Epilepsy, *n* (%)	22 (61.1)	20 (52.6)	.491	36 (60.0)	6 (42.9)	.369
STESS, median [IQR]	2 [1–3]	3 [2–4]	.048	2 [1–3]	3 [3–4]	.013
EMSE, median [IQR]	60 [40–89]	87 [67–105]	.007	70 [45–92]	97 [88–116]	.003
Potentially fatal etiology, *n* (%)	4 (11.1)	17 (44.7)	.002	14 (23.3)	7 (50.0)	.096
Prehospital seizure, *n* (%)	16 (44.4)	12 (31.6)	.338	21 (35.0)	7 (50.0)	.364
Prehospital BDZ, *n* (%)	6 (16.7)	5 (13.2)	.751	8 (13.3)	3 (21.4)	.426
Stroke code, *n* (%)	10 (27.8)	14 (36.8)	.462	20 (33.3)	4 (28.6)	1.000
Focal neurological deficit, *n* (%)	7 (19.4)	10 (26.3)	.584	13 (21.7)	4 (28.6)	.725
Language disturbance, *n* (%)	2 (5.6)	11 (28.9)	.013	13 (21.7)	0 (0.0)	.111
Impaired consciousness, *n* (%)	19 (52.8)	14 (36.8)	.242	26 (43.3)	7 (50.0)	.768
Time to BDZ, min, median [IQR]	167 [77–260]	385 [120–1049]	.033	167 [93–312]	554 [256–1097]	.123
Time to ASM, min, median [IQR]	184 [133–282]	290 [163–644]	.030	205 [136–348]	395 [236–811]	.072
BDZ underdosing, *n* (%)	6 (26.1)	7 (35.0)	.740	9 (25.7)	4 (50.0)	.217
ASM underdosing, *n* (%)	11 (33.3)	21 (55.3)	.094	23 (40.4)	9 (64.3)	.139

*Note*: Timing variables refer to minutes from emergency department arrival.

Abbreviations: ASM, anti‐seizure medication; BDZ, benzodiazepine; EMSE, Epidemiology‐Based Mortality Score in Status Epilepticus; IQR, interquartile range; min, minutes; mRS, modified Rankin Scale; SE, status epilepticus; STESS, Status Epilepticus Severity Score.

Median time to BDZ administration was longer in patients who failed to return to baseline than in those who returned to baseline (385 vs 167 min, *p* = .033), as was median time to ASM administration (290 vs 184 min, *p* = .030). BDZ underdosing was not associated with failure to return to baseline (*p* = .740), whereas ASM underdosing showed a non‐significant trend (*p* = .094). Treatment timing and underdosing variables were not significantly associated with mortality in univariate analysis.

Among patients treated with ASMs, 40 of 71 (56.3%) received ED‐BDZs before ASM and 31 of 71 (43.7%) received ASMs as first‐line treatment. The ASM‐first group was older and had higher premorbid mRS and EMSE score. Failure to return to baseline was similar in the BDZ‐before‐ASM and ASM‐first groups (*p* = .632), as was in‐hospital mortality (*p* = 1.000).

### 3.3 Multivariable analysis

**FIGURE 1:**
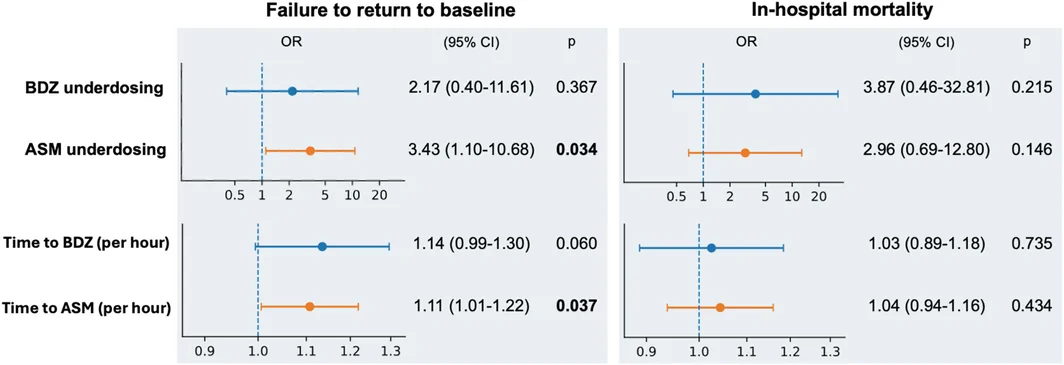
Multivariable associations of treatment features with outcomes. Adjusted odds ratios and 95% confidence intervals (CIs) for failure to return to baseline and in‐hospital mortality. Timing variables are reported per 1‐h increase from emergency department arrival. Models were adjusted for STESS, potentially fatal etiology, and premorbid mRS. Dashed line indicates OR = 1. ASM, anti‐seizure medication; BDZ, benzodiazepine; CI, confidence interval; OR, odds ratio.

Multivariable associations are summarized in Figure 1. Time to BDZ administration showed a trend toward failure to return to baseline (OR 1.14 per hour, 95% CI .99–1.30; *p* = .060), whereas time to ASM administration remained independently associated with this outcome (OR 1.11 per hour, 95% CI 1.01–1.22; *p* = .037). BDZ underdosing was not associated with failure to return to baseline (*p* = .367), whereas ASM underdosing was independently associated (OR 3.43, 95% CI 1.10–10.68; *p* = .034). Time to BDZ and ASM administration, BDZ underdosing, and ASM underdosing were not independently associated with in‐hospital mortality.

## 4 DISCUSSION

In this ED cohort of NCSE, delayed ASM administration and ASM underdosing were independently associated with failure to return to baseline, after adjustment for SE severity, potentially fatal etiology, and premorbid mRS. BDZ underdosing was not associated with functional outcome, and time to BDZ showed a non‐significant trend. No treatment feature was independently associated with in‐hospital mortality. These findings suggest that short‐term functional recovery in ED‐NCSE may be more closely related to timely and adequately dosed ASM treatment, than to BDZ‐related treatment features. Survival, conversely, may be driven mainly by patient‐ and etiology‐related factors.

A major challenge in evaluating NCSE treatment timing is the absence of a reliable onset time.^1^ NCSE onset is frequently unwitnessed, making CSE‐derived time thresholds difficult to apply, especially outside settings with continuous EEG.^1^ We used ED arrival as a pragmatic and reproducible temporal anchor; while this approach might not capture true SE duration, it reflects the diagnostic and therapeutic window in real‐world emergency care.

First‐line ED‐BDZs were administered in ~60% of patients. This deviation from a strict BDZ‐first approach appears to be consistent with registry data showing that NCSE is often managed differently from CSE in clinical practice.^14^ Concern for respiratory depression or prolonged sedation, and fluctuating impairment of consciousness, may explain this behavior.^14^, ^15^ Moreover, prehospital BDZs were recorded separately, because subsequent ED‐NCSE diagnosis indicated that sustained resolution had not been achieved. Whether an additional BDZ trial is beneficial after NCSE confirmation, while clinically relevant, could not be addressed in this cohort.^16^

The absence of an association between BDZ underdosing and outcome should be interpreted cautiously because of sample size and potential confounding by frailty or impaired consciousness. However, this finding is consistent with recent real‐world evidence suggesting that BDZ‐first strategies may not confer a clear prognostic advantage in predominantly NCSE populations.^13^, ^15^ The trend between earlier BDZ administration and better functional outcome may not reflect a direct pharmacological effect of BDZs alone, but rather faster overall management, including earlier EEG, NCSE recognition, and subsequent treatment.^15^

ASM timing and dosing showed a more consistent association with functional outcome. Although shorter time to ASM could also reflect faster overall management, the independent association between ASM underdosing and poor functional outcome suggests a potential treatment‐specific effect. ASMs provide sustained anti‐seizure activity, which may be especially relevant in NCSE with fluctuating ictal activity or IIC patterns.^13^

The lack of association with in‐hospital mortality suggests that treatment features may have less measurable impact on survival when prognosis is mainly driven by etiology, baseline frailty, or systemic illness. As recently suggested, when ictal activity contributes directly to neuronal damage, timely and adequate treatment may be more likely to influence outcome.^17^ Conversely, in NCSE secondary to structural or functional brain dysfunction, prognosis may be less dependent on appropriate treatment.^17^ Unfortunately, due to the small sample size we could not perform etiology‐stratified analyses.

Even among patients with favorable outcome, median treatment delays approached 3 h from ED arrival. This observation suggests that clinically relevant treatment windows in NCSE may be more heterogeneous than in CSE. Treatment delay remains one of the few potentially modifiable components of ED‐NCSE care, and recent data suggest that structured protocols, including seizure‐code strategies, may shorten treatment times and improve hospital outcome.^3^, ^4^ Moreover, recent ED studies suggest that rapid reduced‐montage EEG systems may shorten ED diagnostic latency by identifying nonconvulsive seizures/NCSE or highly epileptiform patterns, supporting their use in emergency pathways.^18^

This study has limitations. Data collection was partly retrospective, which may have introduced information bias, particularly for prehospital treatment. Acute adverse events after ASM loading were not systematically collected, preventing comparison of complications according to ASM dose. Moreover, reduced BDZ or ASM doses may have been clinically justified in some patients. Exploratory comparisons suggested that ASM underdosing was associated with worse premorbid mRS, but not with STESS, EMSE, or potentially fatal etiology, whereas BDZ underdosing was not significantly associated with measured severity variables (Table S2). Nevertheless, residual confounding cannot be excluded.

Sample size limited subgroup analyses by etiology, and mortality analyses were underpowered because of the small number of events. Larger cohorts are needed to assess mortality and clinically relevant subgroups.

ED arrival was used as the temporal anchor, although true NCSE onset was usually unknown. Accordingly, treatment timing reflects ED treatment latency, or door‐to‐treatment time, rather than true NCSE duration.

Few patients required third‐line therapy and NCSE resolution was achieved in nearly all cases, preventing meaningful analyses of treatment escalation.

Finally, because of the observational design and the possibility of residual confounding, these findings should be interpreted as associations rather than evidence of causal effects.

In adults with ED‐NCSE, delayed and underdosed ASM treatment was associated with failure to return to baseline, whereas BDZ‐related variables were not. These findings support a low clinical threshold for suspecting NCSE in the ED and highlight the need for rapid, NCSE‐specific diagnostic and therapeutic pathways.

## AUTHOR CONTRIBUTIONS

F.M. acquired and interpreted the data, planned and designed the study, performed the statistical analyses, and drafted the manuscript. A.Ba., M.F., A.Bo., and L.Z. acquired the data and revised the manuscript. I.F. acquired and interpreted the data, planned and designed the study, and revised the manuscript. S.M. revised the manuscript. All authors read and approved of the final version of the manuscript.

## CONFLICT OF INTEREST STATEMENT

The authors declare no conflicts of interest.

## ETHICS STATEMENT

We confirm that we have read the Journal's position on issues involved in ethical publication and affirm that this report is consistent with those guidelines.

## Supporting information


**Table S1.** Recommended loading doses of benzodiazepines (BDZs) and anti‐seizure medications (ASMs) and thresholds used to define underdosing.
**Table S2.** Patient and nonconvulsive status epilepticus (NCSE) characteristics according to benzodiazepine (BDZ) and anti‐seizure medication (ASM) dose adequacy.

## Data Availability

The data that support the findings of this study are available on request from the corresponding author. The data are not publicly available due to privacy or ethical restrictions.
