# Supplementary material for: Emergency treatment delay and underdosing are associated with poor outcome in non‐convulsive status epilepticus
Source: Epilepsia. 2026 Jul 25;67(9):e163–8. doi: 10.1002/epi.70414 (PMC13592483; doi:10.1002/epi.70414)
Supplement: Supplementary file 1 [file EPI-67-e163-s001.docx]

**Supplementary Material**

**Supplementary Table S1.**

**Recommended loading doses of benzodiazepines (BDZs) and antiseizure medications (ASMs) and thresholds used to define underdosing.**

BDZs and ASMs were classified as underdosed when the administered loading dose was lower than two-thirds of the minimum recommended loading dose according to guideline-based emergency treatment recommendations. When recommended doses were weight-based, thresholds were calculated accordingly.

|  | **Recommended loading dose** | **Threshold for underdosing** |
| --- | --- | --- |
| Benzodiazepines | | |
| Lorazepam | 4 mg (max 8 mg) | < 3 mg |
| Midazolam | 10 mg (max 20 mg) | < 7 mg |
| Diazepam | 10 mg (max 10 mg) | < 7 mg |
| Antiseizure medications | | |
| Lacosamide | 400 mg (max 600 mg) | < 270 mg |
| Levetiracetam | 40–60 mg/kg (max 4500 mg) | < 27 mg/kg |
| Valproate | 20–40 mg/kg (max 3000 mg) | < 15 mg/kg |

**Supplementary Table S2**

**Patient and nonconvulsive status epilepticus (NCSE) characteristics according to benzodiazepine (BDZ) and antiseizure medication (ASM) dose adequacy.**

|  | **Dose adequacy, BDZ** | |  |
| --- | --- | --- | --- |
|  | **Yes (n=30)** | **No (n=13)** | **p** |
| Age, years | 64.5 [55.2–74] | 70 [58–77] | 0.534 |
| Premorbid mRS | 1.5 [0.2–3] | 2 [2–4] | 0.087 |
| STESS | 2 [1–3] | 2 [1–4] | 0.766 |
| EMSE | 58.5 [36–82.8] | 87 [55.5–113] | 0.084 |
| Male sex | 13/30 (43.3%) | 5/13 (38.5%) | 1 |
| Previous epilepsy | 11/30 (36.7%) | 8/13 (61.5%) | 0.185 |
| Potentially fatal etiology | 11/30 (36.7%) | 3/13 (23.1%) | 0.491 |
|  | **Dose adequacy, ASM** | |  |
|  | **Yes (n=39)** | **No (n=32)** | p |
| Age, years | 72 [60.5–77] | 72.5 [63.2–80.2] | 0.323 |
| **Premorbid mRS** | **2 [1–3]** | **3 [2–4]** | **0.026** |
| STESS | 3 [1–3] | 3 [2–3] | 0.564 |
| EMSE | 75 [45–96.5] | 84.5 [63.5–102.8] | 0.286 |
| Male sex | 18/39 (46.2%) | 13/32 (40.6%) | 0.81 |
| Previous epilepsy | 20/39 (51.3%) | 20/32 (62.5%) | 0.471 |
| Potentially fatal etiology | 13/39 (33.3%) | 8/32 (25.0%) | 0.602 |

Data are presented as median [interquartile range] or n/N (%). Continuous variables were compared using the Mann–Whitney U test and categorical variables using Fisher’s exact test. Bold type indicates *p* <0.05.

ASM, antiseizure medication; BDZ, benzodiazepine; mRS, modified Rankin Scale; STESS, Status Epilepticus Severity Score; EMSE, Epidemiology-based Mortality Score in Status Epilepticus.
